# Laparoscopic parastomal hernia repair: keyhole, Sugarbaker, sandwich, or hybrid technique with 3D mesh? An updated systematic review and meta-analysis

**DOI:** 10.1007/s00423-023-03177-9

**Published:** 2023-11-29

**Authors:** Nicos Kritharides, Dimitrios Papaconstantinou, Stylianos Kykalos, Nikolaos Machairas, Dimitrios Schizas, Nikolaos I. Nikiteas, Dimitrios Dimitroulis

**Affiliations:** 1 2nd Department of Propaedeutic Surgery, Laiko General Hospital, Medical School, National and Kapodistrian University of Athens, Ag. Thoma 17, 11527 Athens, Greece; 2 Department of Surgery, General Hospital of Athens «Elpis», 11522 Athens, Greece; 3 3rd Department of Surgery, Attikon University Hospital, National and Kapodistrian University of Athens, 12462 Chaidari, Greece; 4 1st Department of Surgery, Laiko General Hospital, National and Kapodistrian University of Athens, 11527 Athens, Greece

**Keywords:** Parastomal hernia, Laparoscopic, Sugarbaker, Sandwich, Keyhole, Hybrid

## Abstract

**Purpose:**

Parastomal hernia is the most common complication after stoma formation with an incidence that approaches 50% at 2 years postoperatively. In the last decade, different approaches of minimally invasive procedures have been proposed for the treatment of parastomal hernia. Nevertheless, the superiority of one technique over the others remains still unclear. Our objective was to update and systematically analyze current state of research concerning the postoperative outcomes of the four most prevalent minimally invasive techniques.

**Methods:**

A systematic literature search of three databases (Medline, Scopus, Google Scholar) was undertaken for articles published from January 2015 to November 2022. Fifteen studies from a previous meta-analysis on the topic were included.

**Results:**

Thirty-three studies incorporating 1289 total patients were deemed eligible for inclusion in the final analysis. The keyhole technique was associated with the highest incidence of postoperative complications and recurrences (31.3% and 24.1%, respectively), followed by the Sugarbaker technique (27.6% and 9%, respectively). Operative time was among the lowest in patients operated with the 3D mesh technique, while patients undergoing the keyhole technique experienced the shortest cumulative length of hospital stay (6 days).

**Conclusion:**

Each technique demonstrates a unique profile of effectiveness offset by the propensity towards developing postoperative complications. While no conclusive evidence on the optimal technique exist to date, newer minimally invasive techniques show promising results, albeit based on limited data. The future of parastomal hernia repair seems to rely on a highly individualized approach, tailored to the distinctive characteristics of both the hernia and the patient.

## Introduction

Parastomal hernia is the most common complication after stoma formation. According to the European Hernia Society (EHS), a parastomal hernia is defined as “an abnormal protrusion of the contents of the abdominal cavity through the abdominal wall defect created during placement of a colostomy, ileostomy, or ileal conduit stoma” [1, 2]. It is estimated that 30% of patients who undergo stoma creation will develop a parastomal hernia within a year, and about 50% after two or more years of follow-up. A higher incidence is observed in cases of end colostomy, followed by loop colostomy and loop ileostomy [3]. Commonly known risk factors include age (> 60 years), obesity (body mass index (BMI) > 30 kg/m^2^), chronic obstructive pulmonary disease (COPD), malnutrition, use of steroids, emergency surgery, tobacco smoking, postoperative sepsis, and postoperative surgical site infection [4–6]. The predominant symptoms of stomal hernia are pain, bulging, difficulties with stoma device appliance, and skin complications (irritation, erosion). Sporadically, life-threatening situations such as bowel incarceration and strangulation can be observed. Most of these complications can be managed with non-operative measures, with only 30% of patients requiring surgical repair [6, 7].

The most commonly reported approaches for stomal hernia repair include stoma relocation, fascial repair using sutures, and fascial repair using prosthetic mesh with either open or minimally invasive surgery. At present, suture repair for elective surgery is no longer recommended due to high recurrence rates, except in specific circumstances such as strangulation and contamination of the surgical field, where the use of mesh application should be avoided [3]. In the last decade, many minimally invasive procedures have been reported in the literature with varying results. In a previous systematic review in 2015, DeAsis et al. investigated the role of laparoscopic surgery in parastomal hernia repair and concluded that the modified Sugarbaker technique demonstrated superior performance compared to other techniques [8]. Likewise, the sandwich technique showed positive outcomes with low recurrence rates [9]. In another study published in 2015, Szczepkowski et al. described an alternative approach called hybrid with three-dimensional (3D) meshes with promising results [10].

Given an increased number of recently published studies comparing the aforementioned techniques, the objective of this study is to update and systematically analyse the current state of research concerning these techniques and assess the potential superiority of one technique over the others.

## Materials and methods

### Literature search

A systematic literature search of the Medline, Scopus, and Google Scholar databases was undertaken in an effort to identify studies reporting outcomes of surgically treated patients with parastomal hernias for articles published from January 2015 until November 2022. A comprehensive search line was constructed using the terms: “parastomal,” “stomal,” “hernia,” “laparoscopic,” “minimally invasive,” “Sugarbaker,” “Sandwich,” “Keyhole,” “Hybrid,” “3D,” combined with the Boolean operators AND/OR as appropriate for each database. An exhaustive list of abstracts was generated, which after the removal of duplicate studies was screened independently by two authors (NK and DP). All potentially relevant studies were marked for full-text evaluation. The snowballing technique [11] was also employed to manually screen reference lists of selected relevant studies for further articles of interest. Additionally, the fifteen studies in a previous meta-analysis on the topic were also evaluated during this process. The present systematic review and meta-analysis was conducted along PRISMA guidelines [12] and was registered in the International Prospective Register of Systematic Reviews—PROSPERO (ID: CRD42023411640).

### Description of techniques

#### Keyhole technique

The keyhole technique was first described in 1977 by J. Rosin and R. Bonardi. They utilized an onlay mesh with a central slit to cover the hernia defect [13]. In 2003, Hansson et al. adopted the technique in laparoscopic surgery. After adhesiolysis and fascial closure with sutures, an intraperitoneal mesh with a central keyhole of 2 cm is inserted into the peritoneal cavity. It is then fixed around the stoma loop with tacks and sutures, forming a collar around the bowel loop [14]. The main disadvantage of the technique is that the slit by itself is a predisposing factor of hernia recurrence, due to its shrinkage over time [15].

#### Sugarbaker technique

The Sugarbaker technique was described by Paul H. Sugarbaker in 1985. He used an intraperitoneal prosthetic mesh to cover both the fascial defect and a lateralized distal bowel [16]. In 2004, Voitk et al. modified Sugarbaker’s technique and integrated it into laparoscopic approach. After adhesiolysis, an intraperitoneal mesh is inserted into the peritoneal cavity where it is fixed appropriately to cover both the fascial defect and the lateralized distal bowel [17, 18]. Further modifications have been described, such as the use of fascial defect closure before mesh application [19].

#### Sandwich technique

In 2007, Berger et al. introduced the sandwich technique by combining the keyhole and Sugarbaker techniques. The first mesh with the central slit is applied as described for the keyhole technique, followed by a second mesh that covers both the stomal loop and the wall deficiency. The first outcomes from 25 patients were encouraging, with no recurrence in a follow-up period of 12 months [9].

#### Hybrid technique with 3D funnel shape mesh

In 2015, M. Szczepkowski described a novel minimally invasive technique of parastomal hernia repair in 12 patients, the HyPER (hybrid parastomal endoscopic re-do). No recurrences or other complications were reported in a mean period of a follow-up of 13.5 months. The hybrid technique is a combination of laparoscopic and open approach. In the first laparoscopic stage, adhesiolysis is performed, and the stoma bowel is dissected from the fascia. In the second open stage, the hernia sac is visualized and opened, and the bowel stoma is released from the surrounding tissues and passed through the hole of a funnel shape mesh (3D). The mesh is then inserted intraperitoneally, and the fascial defect is closed. The third stage involves reconversion to laparoscopic approach, where the mesh is secured with tacks. The final fourth stage involves maturing of neo-stoma [10].

### Inclusion and exclusion criteria

Following the formulation of the research question, all studies, regardless of publication language, would be considered for inclusion in the final analysis provided that they reported postoperative outcomes of adult patients with parastomal hernias undergoing laparoscopic hernia repair with the Sugarbaker technique, the keyhole technique, the sandwich technique or the 3D mesh technique. The PICO framework was utilized to better delineate the research question as follows: P (adult patients with parastomal hernias), I (minimally invasive parastomal hernia repair), C (none), O (postoperative measures of efficacy). A set of predetermined exclusion criteria was utilized the study piloting process. These exclusion criteria were as follows: (1) case reports, reviews, editorials, opinion articles, and vignettes; (2) studies utilizing open or robotic surgery techniques; (3) small case series incorporating less than 5 total patients; (4) studies with a follow-up shorter than 12 months; and (5) studies with duplicate or overlapping patient populations.

### Data extraction

Two authors (NK and DP) evaluated, in full text, those studies that were deemed potentially eligible by the initial screening process, with a third author (SK) resolving any disagreements during this phase. The predetermined primary outcomes of interest pertained to metrics of postoperative performance for the four investigated techniques, and included recurrence rates, postoperative complication rates, operative time length, and overall length of hospital stay. Secondary outcomes of interest were patient demographics, year of publication, and country of origin of the reported patient cohort.

All data relating to the primary and secondary outcomes of interest were extracted by two authors (NK and DP) and were entered into standardized excel spreadsheets (Microsoft, Redmond, WA, USA) for further tabulation. A third author (SK) oversaw the completeness and accuracy of the data collection process.

### Methodological quality assessment

Each study included in the final quantitative analysis was evaluated for methodological rigorousness using the Newcastle–Ottawa Scale (NOS) [20]. The NOS is an eight-item scale that judges each study based on how representative of the community the patient selection is, how accurate the ascertainment of exposure is, and how objectively the outcome assessment was performed. Scoring results are pooled together to provide a quantitative assessment of the methodological quality of included studies. With 0 representing lowest quality and 9 being the maximum possible.

### Statistical analysis

For the purposes of this analysis, the Open Meta-Analyst software (OpenMeta[Analyst] Software CEBM Brown University, Providence, RI, USA http://www.cebm.brown.edu/openmeta/) was utilized to synthesize individual study data. For categorical variables, cumulative incidence rates were calculated as proportions with corresponding 95% confidence intervals (95% CI), while weighted mean averages and corresponding 95% CI were used to summarily express continuous variables. Due to expected heterogeneity in terms of patient baseline demographics, a random-effects model (DerSimonian and Laird) [21] was a priori selected as the preferred computational method. The Higgins *I*^2^ statistic [22] was employed to quantify observed interstudy statistical heterogeneity as follows; values below 30% represent low heterogeneity, values between 30 and 60% represent moderate heterogeneity, and values above 60% represent substantial heterogeneity. A *p* value equal to or less than 0.05 was considered statistically significant.

## Results

**Fig. 1:**
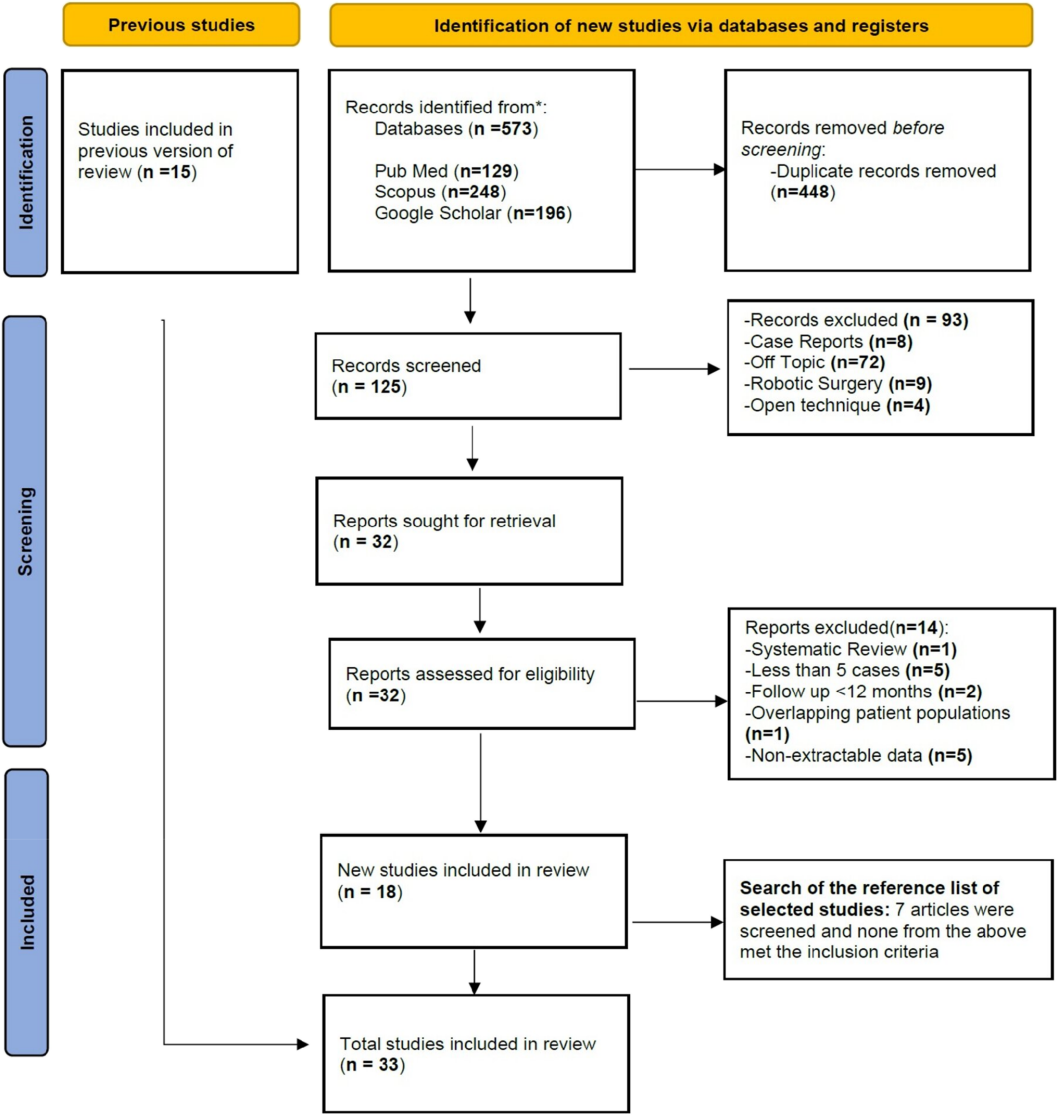
PRISMA flowchart of study selection

After screening 588 unique abstracts and evaluating 47 studies in full text, 33 studies [9, 10, 15, 17, 19, 23–50] incorporating 1289 total patients were deemed eligible for inclusion in the final analysis (Fig. 1) [51]. Overall, 496 (38.5% of the entire cohort) patients underwent laparoscopic parastomal hernia repair with the Sugarbaker technique, 575 (44.6%) patients with the keyhole technique, 125 (9.7%) patients with the sandwich technique, and 93 (7.2%) using 3D mesh technique. Included studies were published from 2004 to 2022 and exhibited geographical variability. Nine studies originated from the USA, sixteen from Europe and eight from Asia. After a mean follow-up that ranged from 12 to 91 months (Table 1), the pooled recurrence rate for all techniques was 13.6%, while overall complication rates were 6.4%. In terms of methodological adequacy, all studies scored in the medium to high range in the NOS scores, with a mean NOS score of 6.8 and a median value of 6 (range 6 to 8).

**Table 1:** Study characteristics and patient baseline demographics. NR, not reported; M, male; F, female; NOS, Newcastle–Ottawa Scale

Author	Country	Age	Sex (M/F)	Number of patients, *n* (%)				Complications	Recurrences n, (%)	Follow-up (months)	NOS score
				Sugarbaker	Keyhole	Sandwich	Hybrid				
Safadi et al. 2004	USA	66	9/0	0	9 (100)	0	0	3	4 (44.4)	24	6
LeBlanc et al. 2005	USA	NR	NR	7 (58.3)	5 (41.7)	0	0	4	1 (8.3)	20	8
Berger et al. 2007	Germany	70	NR	41 (100)	0	0	0	5	8 (19.5)	24	8
Mancini et al. 2007	USA	60	11/14	25 (100)	0	0	0	5	1(4)	19	6
McLemore et al. 2007	USA	66 ± 12	12/7	14 (73.7)	5 (26.3)	0	0	11	0	20	6
Muysoms et al. 2008	Belgium, France	70	13/11	13 (54.2)	11 (45.8)	0	0	2	10 (41.7)	22.3	6
Berger et al. 2008	Germany	69	NR	0	0	47 (100)	0	4	1(2.1)	20	8
Hansson et al. 2008	Netherlands	63	27/28	0	55 (100)	0	0	31	20 (36.3)	36	8
Craft et al. 2008	USA	66	NR	16 (76.2)	5 (23.8)	0	0	10	1 (4.8)	14	6
Pastor et al. 2009	USA	60 ± 5.13	5/7	9 (75)	3 (25)	0	0	4	4 (33.3)	13.9 ± 4.5	6
Jani et al. 2010	India	68.9	7/2	0	9 (100)	0	0	2	0	12.6	8
Wara et al. 2011	Denmark	62	36/36	0	72 (100)	0	0	30	2(2.8)	36	8
Mizrahi et al. 2012	UK	63.5	10/19	0	29 (100)	0	0	5	13 (44.8)	28	6
Asif et al. 2012	USA	61.6	20/13	14 (42.4)	19 (57.5)	0	0	13	11 (33.3)	35.8	6
Hansson et al. 2013	Netherlands, Spain, Germany, Belgium	63	40/21	61 (100)	0	0	0	12	4 (6.6)	26	6
DeAsis et al. 2015	USA	64.3	23/20	25 (58.1)	18 (41.9)	0	0	29	15 (34.9)	18.1	6
Szczepkowski et al. 2015	Poland	71	9/3	0	0	0	12 (100)	1	0	13.5	8
Köhler et al. 2015	Austria	68.4 ± 26.1	NR	4 (6.3)	22 (34.9)	21 (33.3)	16 (25.4)	11	13 (20.6)	30	6
Zhang et al. 2016	China	69.9 ± 8.8	NR	0	0	0	16 (100)	0	0	16.6 ± 8.8	6
Levy et al. 2016	USA	64 ± 10	6/14	20 (100)	0	0	0	9	1(5)	16.7	6
Fischer et al. 2017	Austria	64.8 ± 27	NR	0	0	0	41 (100)	NR	3(7.3)	38	6
Oma et al. 2017	Denmark	70.3	NR	63 (87.5)	9 (12.5)	0	0	NR	7(9.7)	12	8
Rajapandian et al. 2018	India	37	NR	0	22 (100)	0	0	7	1(4.5)	23	8
Yan et al. 2018	China	65 ± 1	40/25	0	65 (100)	0	0	8	1(1.5)	29 ± 2.1	8
Huang et al. 2018	China	65 ± 7.4	5/3	8(100)	0	0	0	2	0	13	6
Hashida et al. 2019	Japan	76	5/8	0	0	13 (100)	0	0	0	36	6
Olmi et al. 2019	Italy	68	37/53	0	90 (100)	0	0	4	4(4.4)	12	7
Rege et al. 2019	India	NR	8/6	14 (100)	0	0	0	1	0	NR	6
Bertoglio et al. 2020	Italy	70.5	18/14	0	19 (59.3)0	13 (40.6)	0	6	4 (12.5)	36.5	6
Gameza et al. 2020	Denmark	62.5	64/71	61 (45.1)	74 (54.8)	0	0	51	11 (8.1)	34	8
Mäkäräinen-Uhlbäck et al. 2021	Finland	67.5	NR	68 (57.6)	11 (9.3)	31 (26.2)	8 (6.7)	53	30 (25.4)	39	8
Suwa et al. 2021	Japan	72	16/17	33 (100)	0	0	0	3	3(9.1)	48.5	8
Laycock et al. 2022	UK	70	NR	0	23 (100)	0	0	4	2(8.7)	91	6

### Recurrence rates

**Fig. 2:**
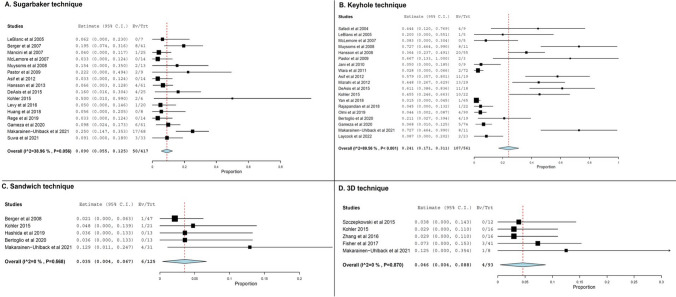
Forest plots of recurrence rates for various techniques

Parastomal hernia recurrence rates were the most commonly reported outcome, with 16 studies reporting on recurrence rates after the Sugarbaker technique, 19 after the keyhole technique and 5 studies for the sandwich and 3D mesh techniques (Table 2). The keyhole technique was associated with the highest incidence rate amongst the evaluated techniques (24.1%, 95% CI 17.1 to 31.1%, Fig. 2B), with substantial interstudy statistical heterogeneity being present in the analysis (*I*^2^ = 89.6%). The technique described by Sugarbaker was observed to be second in terms of recurrence rate (9%, 95% CI 5.5 to 12.5%, Fig. 2A) with moderate statistical interstudy heterogeneity noted (*I*^2^ = 38.9%). A substantially smaller number of studies reported recurrence rates with the sandwich and 3D mesh techniques (Table 2), which were found to be among the lowest ones reported (sandwich technique; 3.5%, 95% CI 4 to 6.7%, Fig. 2C, and 3D mesh technique; 4.6%, 95% CI 4 to 8.8%, Fig. 2D), with the reported results being uniform in terms of statistical heterogeneity (*I*^2^ = 0%).

**Table 2:** Summary outcomes for the different methods of minimally invasive parastomal hernia repair techniques. *I*^2^, the Higgin’s statistic

Outcome	Number of studies	Total patients	Effect size	95% Confidence intervals	*I*^2^	*p* value
Subarbaker						
Complications (%)	13	381	27.6	18.2–37.1	78.5	< 0.001
Recurrences (%)	16	417	9	5.5–12.5	38.9	0.05
Operative time (min)	5	170	165.8	137.7–193.9	89.3	< 0.001
Length of hospital stay (days)	6	195	9.6	5.7–13.5	96.9	< 0.001
Keyhole						
Complications (%)	16	542	31.3	20.1–42.6	91	< 0.001
Recurrences (%)	19	561	24.1	17.1–31.1	89.6	< 0.001
Operative time (min)	7	235	144.2	83.2–205.3	99.9	< 0.001
Length of hospital stay (days)	6	209	6	4.1–7.9	95.6	< 0.001
Sandwich						
Complications (%)	5	125	13.2	3.7–22.6	66	0.01
Recurrences (%)	5	125	3.5	4–6.7	0	0.56
Operative time (min)	2	78	130.1	118.3–141.9	0	0.72
Length of hospital stay (days)	2	78	14.5	0–30.8	97.4	< 0.001
3D mesh technique						
Complications (%)	4	52	6	0–12.7	0	0.42
Recurrences (%)	5	93	4.6	0.4–8.8	0	0.87
Operative time (min)	2	24	144	99.1–188.9	98.9	< 0.001
Length of hospital stay (days)	2	8	8.4	5.1–11.7	81.8	0.01

**Fig. 3:**
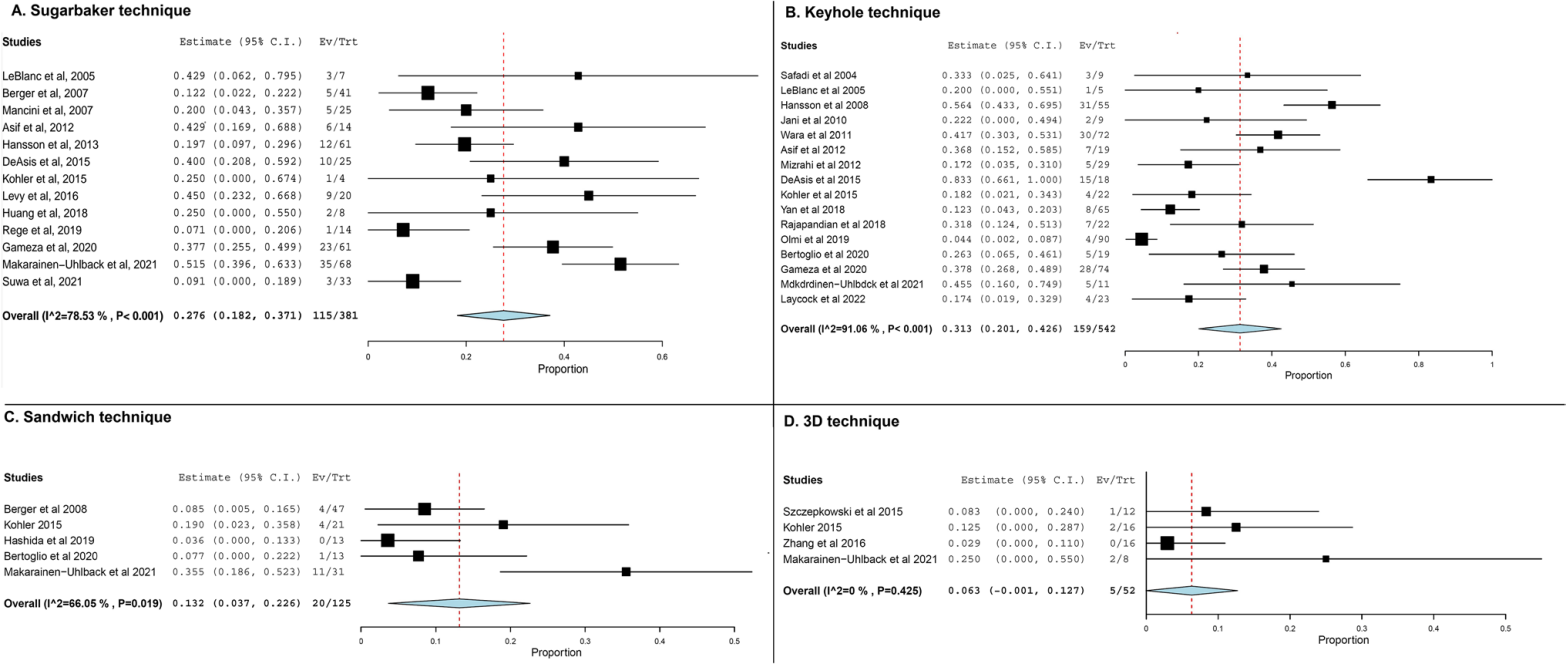
Cumulative complication rates for investigated techniques

### Postoperative complication rates

Complication rates were the second most commonly reported outcome, with 13 studies reporting complications after Sugarbaker technique, 16 after keyhole technique, 5 following the sandwich technique, and 4 after 3D mesh repairs (Table 2). Specifically, the highest incidence of postoperative complications was noted after repairs with the keyhole technique (31.3%, 95% CI 20 to 42.6%, Fig. 3B), followed by the sandwich technique (13.2%, 95% CI 3.7 to 22.6%, Fig. 3C), the Sugarbaker technique (27.6%, 95% CI 18.2 to 37.1%, Fig. 3A), and lastly the 3D mesh technique (6.3%, 95% CI 0 to 12.7%, Fig. 3D). Statistical heterogeneity was substantial amongst studies reporting the keyhole (*I*^2^ = 91%) and sandwich (*I*^2^ = 66%) techniques, moderate in studies involving the Sugarbaker technique (*I*^2^ = 38.9%), and nonexistent amongst studies reporting on the 3D mesh technique. In Table 3, we present specific complications that were described in the majority of studies, such as surgical site infections (SSIs), mesh infection, bowel obstruction, postoperative ileus, and other complications (including cardiopulmonary complications).

**Table 3:** Specific complications that recorded in different studies

Technique	SSI (surgical site infection)	Mesh infection	Bowel obstruction	Postoperative ileus	Other complications*
Keyhole (*n* = 575)	19 (3.3%)	5 (0.9%)	11 (1.9%)	23 (4%)	103 (18%)
Sugarbaker (*n* = 496)	30 (6%)	8 (1.6%)	13 (2.6%)	15 (3%)	37 (7.45%)
Sandwich (*n* = 125)	8 (6.4%)	0	2 (1.6%)	0	10 (8%)
Hybrid (*n* = 93)	1 (1%)	0	1 (1%)	0	3 (3.2%)

*n* number of patients

*Any adverse event that not included in the described complications (as cardiopulmonary complications)

### Operative time

**Fig. 4:**
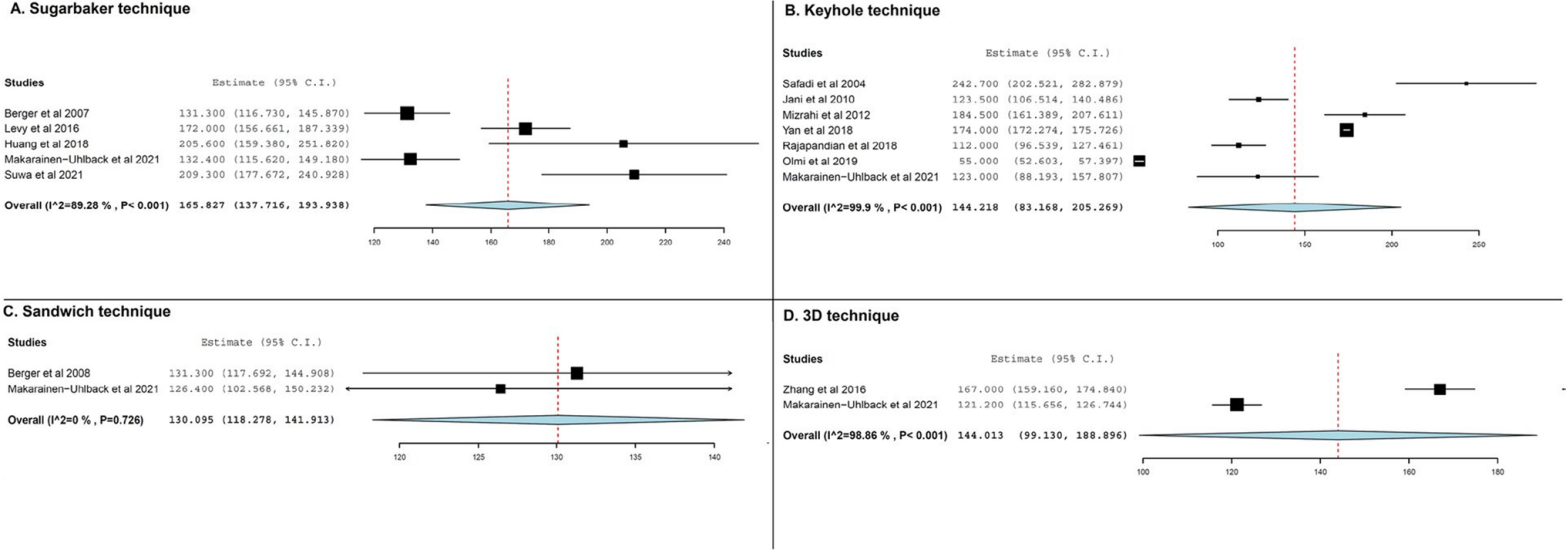
Weighted mean operative time length (in minutes) for various techniques

The subset of studies that reported on operative time length was comparatively smaller, with 5 studies recording operative times for the Sugarbaker technique, 7 for the keyhole technique, and 2 studies for each of the sandwich and 3D mesh techniques (Table 2). The longest average operation times were registered with the Sugarbaker technique (165.8 min, 95% CI 137.1 to 193.9, Fig. 4A) followed by the keyhole technique (144.2 min, 95% CI 83.2 to 205.3, Fig. 4B). In both cases, substantial interstudy heterogeneity was observed (*I*^2^ = 89.3% and 99.9%, respectively). Concerning the sandwich and 3D techniques, operative times were the shortest with the former (130.1 min, 95% CI 118.3 to 141.9, Fig. 4C), while operative times associated with the 3D technique were comparable to those observed with the keyhole technique (144 min, 95% CI 99.1 to 188.9, Fig. 4D).

### Length of hospital stay

**Fig. 5:**
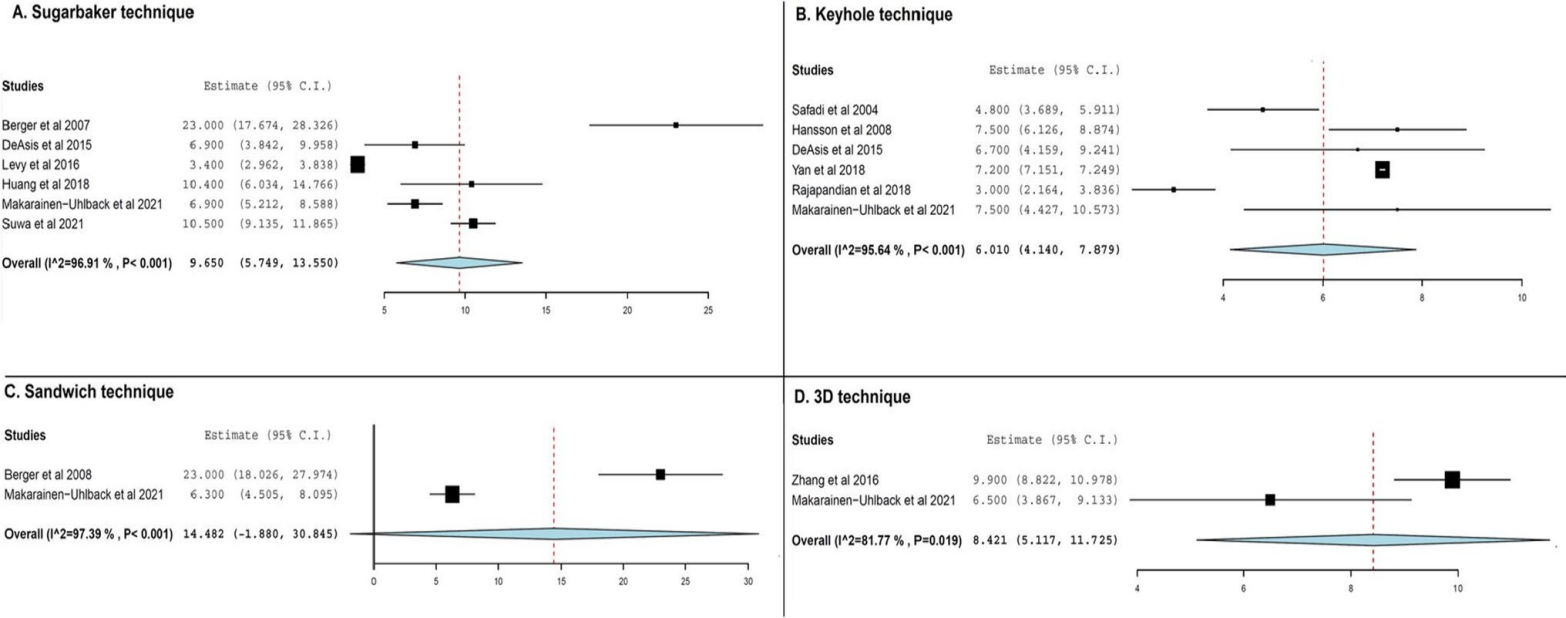
Pooled average length of hospital stay in various operative techniques

The duration of postoperative hospital stay was reported in 6 studies concerning the Sugarbaker and keyhole techniques, with another two presenting relevant data on the sandwich and 3D techniques. Pooled results indicated that the keyhole technique had the shortest length of hospital stay (6 days, 95% CI 4.1 to 7.9, Fig. 5B), while longer hospitalization was observed in the Sugarbaker technique group of patients (9.7 days, 95% CI 5.8 to 13.6, Fig. 5A). With regard to the sandwich and 3D mesh techniques, the average postoperative length of hospital stay was 14.5 days (95% CI 0 to 30.85 days) and 8.4 days (95% CI 5.1 to 11.7 days), respectively. Statistical heterogeneity was substantial in all analyses (*I*^2^ ranging from 81.8 to 97.4%, Table 2).

## Discussion

The primary finding of the current systematic review is that the novel approaches, sandwich, and hybrid with 3D meshes, demonstrate superior outcomes in terms of recurrences when compared to the keyhole and Sugarbaker techniques (3.5% and 4.6% versus 24% and 9%, respectively). Notably, the keyhole technique is associated with the highest recurrence rates (24.1%) and postoperative complications (31.3%), consistent with the results of the previous systematic review by DeAsis et al. [8]. The sandwich technique demonstrates the lowest recurrence rates (3.5%), but it is accompanied by a high rate of postoperative complications (13.2%), followed by the hybrid technique with a recurrence rate of 4.6% and the lowest postoperative complication rates (6.3%). The keyhole technique exhibits the highest recurrence rates but has among the shortest operation times, while the Sugarbaker technique presents an acceptable recurrence (9%) and a moderately increased complication rates (27.6%).

The second noteworthy finding in this updated systematic review is the lower overall recurrence rate compared to the previous meta-analysis in 2015 [8] In their study in 2015, DeAsis et al. reported an overall recurrence rate of 17.4%, with 10.2% (95% CI:3.9–19.0) for Sugarbaker and 27.9% (95% CI: 12.3–46.8) for keyhole [8]. These rates were higher than what we observed in our current review, which showed an overall recurrence rate of 13.6% with 24.1% for keyhole and 9% for Sugarbaker. The development and advancement of these techniques in recent years may be partly responsible for the improved outcomes. Recent studies have indicated that fascial closure with interrupted sutures before mesh application is a modification that leads to lower recurrence rates [15, 19]. Olmi et al. also introduced a modification to the keyhole technique involving fascial closure and stoma fixation in defect edges before applying the mesh. The results of this adjustment in 90 patients led to only 4 recurrences during the follow-up. According to the authors, their adaptations achieved a recurrence rate as low as that of the Sugarbaker technique [15].

In addition to the aforementioned technique adjustments, recent studies emphasize the essential role of mesh material choice in reducing hernia recurrences. De Asis et al.’s systematic review revealed that many included studies used ePTFE (extended polytetrafluoroethylene) mesh, characterized by its microporous nature and propensity for shrinkage [8]. However, in studies conducted after 2015, most authors preferred monofilament polyester mesh with a collagen film barrier or 3D funnel-shaped meshes made of polyvilidene fluoride (PVDF) and polypropylene. These materials promote superior tissue-mesh integration, contributing to a reduction in mesh shrinkage, particularly in procedures like the keyhole technique, resulting in decreased recurrences [10]. Finally, the expertise of specialized surgeons, the evolving understanding of parastomal hernia formation, and the identification of key risk factors for hernia recurrences have all contributed to the optimal results of the last decade.

Despite the lower recurrence rates in our updated review, we have observed significantly higher overall postoperative complication rates compared to those reported by DeAsis et al. (6.4% vs 1.8%) [8]. This difference primarily arises from our expanded definition of complications. Due to the high variability among studies and the lack of precise data on postoperative complications we chose to categorise any postoperative adverse events, as postoperative complications. Nevertheless, to maximize the impact of our findings we separately recorded specific complications, such as surgical site infections, mesh infections, bowel obstruction, and postoperative ileus, as outlined in the “Results” section. Consequently, by documenting all adverse events—ranging from postoperative paralytic ileus to cardiopulmonary complications, some of which pertain to the same patient—we increased the postoperative complication rates.

Another noteworthy point is the comparison of the two most prevailing techniques namely the keyhole and Sugarbaker techniques. In our study, we observed a significant difference in recurrence rates, highlighting the superiority of the Sugarbaker technique over the keyhole (9% versus 24.1%). A.M Fleming et al. conducted a recent systematic review of studies comparing only keyhole and Sugarbaker techniques (both open and laparoscopic), but they failed to demonstrate a superiority of one technique over the other. In their initial overall analysis, they observed that the modified Sugarbaker technique had lower recurrence rates compared to the keyhole technique. Nevertheless, in their subgroup analysis (studies after 2015), they observed that both techniques demonstrated similar results in terms of recurrences. According to the authors, several factors may have contributed to this observation, including the evolution of keyhole technique and the development of modern mesh materials. Additionally, there were differences in the study populations between studies conducted before and after 2015, as most studies after 2015 were conducted in Europe, whereas studies before 2015 were mainly conducted in the USA [52].

Thus, the question that arises is, 'What is the preferable technique for laparoscopic parastomal hernia repair?'. Li Luan et al. designed an algorithm to determine which is the technique of choice for the treatment of recurrent parastomal hernias. Firstly, the authors used laparoscopy to investigate the presence of infection, adhesions, or tumor recurrence. In case of infection, they proceeded to simple suture repair. In the presence of any adhesions, they categorized them as light, medium, and heavy. In the presence of light adhesions with a short bowel loop, they proceeded to keyhole technique, while in the case of a long bowel loop, they preferred the Sugarbaker approach. For medium adhesions and bowel injury they performed onlay mesh repair, but in the absence of bowel injury, they used laparoscopic re do with or without keyhole/Sugarbaker technique. Finally, in the case of heavy adhesions they favored onlay repair. The application of this algorithm resulted in zero recurrences on a mean follow-up of 32.8 ± 3.77 months, encompassing a total of 17 cases [53]. A similar therapeutic algorithm, as described above, will facilitate future studies in the objective evaluation of the described techniques and clarify their outcomes in distinctive circumstances.

Another issue we need to acknowledge is the role of prophylactic mesh during ostomy creation. Is the principle “prevention is better than cure” applicable in stomal hernia? Current European Hernia Society guidelines strongly recommend the usage of prophylactic mesh during permanent end stoma creation, to decrease the incidence of parastomal hernias [3]. The initial results of a recent meta-analysis of randomized controlled trials that compared the use or not of prophylactic mesh placement during end colostomy construction ally with the EHS statement [54]. However, in a subgroup meta-analysis of the studies conducted the last 5 years, the authors failed to detect a statistically significant difference in parastomal hernia prevalence after prophylactic mesh application. As the authors suggest, these results could be attributed to changes in the patient population. Nowadays, patients are more prone to obesity, suffer from many comorbidities and are regularly exposed to neoadjuvant treatments, factors that affect tissue healing mechanisms and predispose to hernia formation. Therefore, although the use of prophylactic mesh may contribute to a decline in hernia formation, this potential benefit needs further investigation [54].

Moreover, it is fundamental to clarify the potential superiority of extraperitoneal route of stoma creation over the intraperitoneal route. In the 2018 EHS guidelines, authors argued that making a recommendation on this topic was ambiguous due to the lack of randomized controlled trials [3]. In 2022, Luo et al. conducted to a meta-analysis of randomized controlled trials comparing transperitoneal and extraperitoneal colostomy to analyze the outcomes of each technique. The meta-analysis results showed that extraperitoneal colostomy demonstrated a lower incidence of parastomal hernia and parastomal prolapse, accompanied by higher rates of defecation sensation. Defecation sensation, refers to the stimulation of parietal peritoneum’s nerves that occur during stool passage through the bowel lumen in extraperitoneal colostomy. Patients may occasionally establish a level of defecation control due to abdominal muscle contractions, thereby improving their quality of life. Remarkably, extraperitoneal colostomy appears as a promising technique for hernia prevention. Further controlled studies comparing prophylactic mesh with extraperitoneal colostomy creation are essential to determine the most appropriate prevention method [55].

Another crucial issue necessitating clarification is the management of concomitant incisional hernias alongside parastomal hernias. Reported incidence rates vary widely, ranging from 13 to 58.3% [10, 48]. The European Hernia Society classifies parastomal hernias into four types based on defect size and the presence of concomitant incisional hernias [1]. A comprehensive literature review regarding the most suitable minimally invasive surgical approach in these cases failed to yield specific recommendations. To shed light on this issue, we examined various studies to identify the surgical approaches employed in such cases. Köhler et al. used a second intraperitoneal flat mesh to cover the midline incisional hernia in the hybrid technique [36]. Other authors employed the same mesh to cover both hernia defects in Sugarbaker and sandwich approaches [19, 27, 43]. Lambrerht used transversus abdominis muscle release (TAR) combined with the modified Sugarbaker technique for distal incisional hernias, whereas midline incisional hernias required enhanced-view Rives-Stoppa (eRS) technique [56]. Regarding the recurrence rates, Gameza et al. discovered no significant differences after simultaneously repairing parastomal and concomitant incisional hernias [46]. The information mentioned above relies on individual centers’ experiences, and there is a noticeable absence of standardized recommendations in this regard. Future studies should encompass a more comprehensive exploration, aiming to evaluate the efficacy of each technique and their applicability in cases involving concomitant incisional hernias.

Several inherent limitations of our study should be acknowledged. This systematic review is mainly limited to observational studies, with the majority being retrospective case series studies. Many of the included studies did not provide sufficient data on parastomal hernia classification, patient characteristics, risk factors for hernia formation and recurrence, urgency of surgery, and criteria for accurately diagnosing hernia recurrence. In addition, morbidity rates were seldom reports and thus the present analysis relied on evaluating pooled complication incidence rates, which lack a clear estimation of severity since relevant Clavien-Dindo scores were not provided. The encountered heterogeneity in terms of study population and outcome reporting makes it challenging to compare the different techniques, and it limits the overall generalizability of the findings presented herein. Further prospective, well-designed trials, with clearly set definitions and uniform outcome reporting are essential for exploring the exact efficacy of each technique and how it fits within the current cadre of minimally invasive approaches for managing parastomal hernias.

## Conclusion

While minimally invasive surgery for parastomal hernia repair is now a reality, the technique of choice remains a subject of debate. This systematic review reinforces previous observations that the novel techniques “sandwich” and “hybrid with 3D mesh” appear to offer superior outcomes in terms of recurrences and safety profiles compared to “keyhole” and “Sugarbaker” techniques. Notably, over the past decade, there has been a decline in the overall recurrence rate for all techniques, possibly due to modifications such as fascial closure, surgeons’ experience in minimally invasive surgery, and advancements in mesh materials. Based our results and recent studies in the field, it appears that a well-designed, individualized approach that considers preoperative diagnosis, preoperative hernia classification, intraoperative findings, and decision-making models is likely to become the gold standard for the minimally invasive treatment of parastomal hernias in the future.

## Data Availability

The data that support the findings of this study are available on request from the corresponding author.
